# Short-term renewable energy consumption and generation forecasting: A case study of Western Australia

**DOI:** 10.1016/j.heliyon.2022.e09152

**Published:** 2022-03-22

**Authors:** Bilal Abu-Salih, Pornpit Wongthongtham, Greg Morrison, Kevin Coutinho, Manaf Al-Okaily, Ammar Huneiti

**Affiliations:** a The University of Jordan, Jordan; b The University of Notre Dame Australia, Australia; c Curtin University, Australia; d Rewalty Pty Ltd, Australia; e Jadara University, Jordan

**Keywords:** Energy consumption, Energy generation, Renewable energy, Time series forecasting, Peer-to-peer energy trading

## Abstract

Peer-to-Peer (P2P) energy trading has gained much attention recently due to the advanced development of distributed energy resources. P2P enables prosumers to trade their surplus electricity and allows consumers to purchase affordable and locally produced renewable energy. Therefore, it is significant to develop solutions that are able to forecast energy consumption and generation toward better power management, thereby making renewable energy more accessible and empowering prosumers to make an informed decision on their energy management. In this paper, several models for forecasting short-term renewable energy consumption and generating are developed and discussed. Real-time energy datasets were collected from smart meters that were installed in residential premises in Western Australia. These datasets are collected from August 2018 to Apr 2019 at fine time resolution down to 5 s and comprise energy import from the grid, energy export to the grid, energy generation from installed rooftop PV, energy consumption in households, and outdoor temperature. Several models for forecasting short-term renewable energy consumption and generating are developed and discussed. The empirical results demonstrate the superiority of the optimised deep learning-based Long Term Short Memory (LSTM) model in forecasting both energy consumption and generation and outperforms the baseline model as well as the alternative classical and machine learning methods by a substantial margin.

## 1 Introduction

The ongoing growth of the world's population has led to a dramatic increase in energy demand which is expected to approach a 25% increase, in comparison to the actual consumption, by 2040 [1]. To meet such demand, efforts have been consolidated toward developing advanced renewable energy systems that have led to establishing new economic alternatives [2, 3]. In this context, sharing economy has exploded in popularity over recent years and is expecting to continue its trend and expand to many market sectors e.g., Peer-to-Peer (P2P) transportation (Uber), P2P accommodation (Airbnb), P2P energy trading, etc. Supply and demand are matched using high-speed ICT in sharing economy which is disrupting traditional business models. In the energy sector, the development and the widespread deployment of smart meters offer the essential infrastructure for sharing economy through P2P energy trading [4]. This leads to affordable clean energy and ultimately to the sustainable energy transition. Further, this commences venues for power system markets as well as potentially allowing a transformation to customers sharing electricity. In addition, P2P electricity markets may allow investing in locally produced renewable energy which provides more flexibility for consumers to choose their source of electric energy. A sharing economy in the energy sector can be seen through a P2P local energy trading market allowing energy buyers and sellers to trade with each other bypassing the central system.

The Australian Government has funded a research project (RENeW Nexus project [5]) to investigate the effect of using blockchain technology as well as data analytics to enable P2P trading of energy and water. In the first phase of the project, electric smart meters were deployed in fifty houses across the city of Fremantle, Western Australia. The energy data was collected in real-time and included: energy data imported from the grid; energy data exported to the grid; energy data generated from the installed rooftop PV; and energy consumed at the households. To enable effective P2P energy trading, real-time forecasting is essential to estimate the likely energy usage and production, forming crucial insights that can substantially increase operation efficiency and make the most efficient energy trading between prosumers and consumers. In a traditional centralised power supply, consumers purchase electricity from utilities and eligible counterparts in fixed tariffs. On contrary, consumers become prosumers (i.e., they can consume as well as produce power) in P2P energy trading and their role can be smoothly switched, thereby allowing to either purchasing power or selling it to achieve a win-win in an open market model [6]. However, decision-making, including electricity price bidding, energy consumption and generation scheduling, etc., in such a decentralised environment is not an easy task; this is due to the complexity, independency, uncertainty, and unpredictability of the market in reality [7, 8]. Further, reliability analysis, security assessment, voltage excursion, thermal overloading, and other network constraints [9, 10, 11, 12, 13] augment the trading complexity. Therefore, real-time energy forecasting is an essential guide for buyers and sellers to assist the bidding process on the spot market, to a better understanding of real-time prosumers behaviour, and to enable peak-shaving and smooth power dispatching [14, 15, 16]. It also allows to attain security of operations conducted under tight reserve margins [17].

In addition, a traditional highly centralised energy management and market is controlled by a few key players. A transition to a more democratic decentralised market is needed. The real-time forecasting for P2P energy consumption and generation is not only enabling the transition but also providing a solution to the phenomenon known as the utility death spiral [18]. This global phenomenon is caused by the increased adoption of rooftop photovoltaic (PV) panels which reduces overall grid electricity demand without affecting peak demand. With fewer paying customers to maintain the necessary infrastructure, utilities are required to increase electricity prices; this in turn encourages greater adoption of PV panels. This is exacerbated by the decreasing costs of PV technology. Battery adoption by PV users can help to reduce peak demand however excess electricity is still exported to the grid in an inefficient manner, at times when demand is low. A study of actual P2P trading system has been reported and published in [4].

In this paper, short-term forecasting of energy consumption and production using actual data is explored. Real-time data were collected from smart meters that were installed in residential premises and used to evaluate the efficacy and effectiveness of statistical and machine learning techniques. Well-known classical (statistical) and AI-based models are used to analyse energy consumption and production. The comprehensive comparison is investigated and illustrated. The following are the key contributions of this paper:

• A two-fold analytical model to forecast energy consumption and generation in P2P settings is designed and implemented.
• An auto-ML model and various classical and advanced AI-based techniques are incorporated, and their utility is evaluated and compared comprehensively.
• To the best of our knowledge, this is the first paper that furnishes a forecasting model for both energy consumption and generation based on real data captured from a P2P grid system in the state of Western Australia.

The rest of this paper is organized as follows: Section 2 offers a review of various intelligent techniques used in energy consumption and generation. Section 3 discusses different forecasting approaches used for time series analysis. Section 4 provides a comprehensive discussion on the models’ development. The carried out experiments to validate the utility of the model are presented and discussed in Section 5. Section 6 discusses the empirical results and point to the limitations of this study. Section 7 concludes the paper.

## 2 Related works

The application of Artificial Intelligence (AI) has extended to numerous industrial domains. This is due to the sophisticated architecture of AI algorithms that empowers them to address a wide variety of real-life problems [19, 20, 21, 22, 23]. Forecasting of energy consumption and generation is an important venue of research that is used to tackle related issues such as demand response, fault detection and troubleshooting, model predictive control, and energy management and optimization [24]. Hence, renewable energy forecasting has attracted a great deal of interest recently [25, 26, 27, 28, 29]. Energy forecasting techniques are commonly categorized into two key approaches, namely conventional models and AI-based models. In a recent survey [30], it was indicated that the proportion of AI-based models to conventional/classical models, based on a study of 128 models, are 48% and 43%, respectively. This implies the significance of both models in the designated task even though AI-based models are much more sophisticated. In fact, some classical models, under certain assumptions, overshadow advanced AI approaches [31]. In this section, we visit studies that have been carried out in the area of both energy forecasting and consumption using the aforementioned approaches.

In terms of energy consumption forecasting, various studies were proposed to tackle this issue incorporating both classical models [32, 33, 34, 35, 36, 37, 38] and AI-based models [36, 37, 38, 39, 40, 41]. For example [32], proposed a novel time-series smoothing model for medium-term electricity consumption forecasting. The model is based on a semi-parametric model that offers an extension of the semi-parametric and nonparametric vector autoregression model. Long-term energy consumption forecasting was addressed in [33] where authors combined a mixed data sampling model with an autoregressive distributed lag to predict energy demand in China. For energy consumption in Turkey and Pakistan, Akpinar et al. [34] and Hussain et al. [35] applied the ARIMA model for this designated task respectively. Various other conventional models have been incorporated for energy consumption prediction, including logistic regression [36], linear regression [37], and Nonlinear regression (NLR) [38].

Popular AI-based approaches such as Artificial Neural Networks (ANN), Support Vector Machine (SVM), and Random Forest (RF) were also utilized in energy forecasting models. ANN includes various machine learning algorithms that were used in the literature. For example, three layers of Feedforward Neural Network (FFNN) are used in [42]. An optimized hybrid algorithm that uses FFNN was presented by [39] to predicted short-term building energy forecasting. Another optimization algorithm, for short-term energy consumption, was proposed which incorporates FFNN and Bayesian regularization algorithm [40]. Another thread of efforts adopts Deep Learning (DL) techniques leveraged by advanced specifications of modern computers. DL models are mainly represented by the recurrent neural network (RNN) to forecast energy consumption [43]. He [41] incorporated RNN to model the implicit dynamics and obtain the predicted load along with CNN that was used in their work to extract significant features from the historical load sequence. Long Short-Term Memory (LSTM) is a special type of RNN that has been proven to outperform modern models [41, 44]. Authors of [45] employed LSTM architecture toward electricity consumption forecasting. They presented the sequence-to-sequence architecture to obtain a random number of formerly attainable load measurements and use them as input to approximate the load for future time steps.

Statistical models using both classical models as well as AI-based models were utilised to predict energy generation, including ARMAX models [46], ARIMA/SARIMA models [47, 48, 49], multiple regression [50], regression with neural networks [51, 52, 53], support vector machine (SVM) [54], etc. For example, the authors of [55] transformed time series data into stationary data using the ARIMA model. Their model for predicting energy is validated using the Akaike Information Criterion (AIC) and Residual Sum of Squares (SSE). Ayub et al. [56] applied SVM with three parameters (kernel parameter, cost penalty, and incentive loss function parameter) on the electricity load data set. Using various machine learning methods was also reported on a real data set collected in France [57]. Li [58] introduced short-term solar irradiance forecasting algorithms based on Hidden Markov Model and SVM regression. The paper presents an approach to predict Photovoltaic (PV) generation under different weather conditions. Statistical and intelligent models based on machine learning used for PV generation forecasting were also reported in [3, 59, 60, 61].

Table 1 demonstrates a summary of several approaches for forecasting energy generation and consumption. As illustrated in the table, most of the current studies were carried out using limited variations of statistical techniques to predict either energy generation or energy consumption. Also, few attempts were undertaken to conduct very short-term energy forecasting. Implementing short-term energy forecasting approaches benefits real-time or near real-time energy dispatching systems, and it has proven utility in providing better coordination of resources [14]. Our approach, on the other hand, discusses the implementation of short-term (i.e., hourly) power generation as well as power consumption models using a variety of classical and sophisticated AI-based techniques. Further, to the best of our knowledge, this is the first paper that offers a two-fold energy consumption and generation model based on real data captured from a P2P grid system in the state of Western Australia.

**Table 1:** A Summary comparison between various energy generation and consumption forecasting methods.

Ref.	Forecasting Model	Consumption/Generation	Data Source	Data Temporality	Forecasting Horizon	Country	Evaluation Metric(s)
[32]	Vector Autoregression	Consumption	Suzhou Municipal Bureau of Statistics	Jan 2004 to Jan 2014	Monthly	China	MAPE
[33]	ADL-MIDAS	Consumption	National Bureau of Statistics of China	2016	Quarterly	China	RMSFE
[34]	ARIMA	Consumption	Adapazari Natural Gas Distribution	2009 to 2012	Monthly	Turkey	MAPE
[35]	Holt-Winter and ARIMA	Consumption	Pakistan Economic Survey	1980 to 2011	Annually	Pakistan	RMSE, MAPE
[62]	ARMA + Kalman filter	Consumption	Hellenic Public Power Corporation S.A.	Jan 2004 to Dec 2006	Daily	Greece	MAPE
[63]	MA + SARIMA + PSO	Consumption	Power Grids of China	Dec 2003 to Dec 2009	Monthly	China	MAE, RMSE, MAPE
[64]	Exponential smoothing model + Bayesian inference	Consumption	IEA website	1990 to 2014	Yearly	Japan	AAEP
[65]	FARX	Consumption	Residential Energy Consumption Survey	Apr 2018 to Jul 2015	Hourly	USA	MAPE, RMSE
[36]	Multi-cycle logistic model	Consumption and Generation	US Energy Information Agency	1949 to 2015	Yearly	USA	R-square
[66]	Holt-Winters exponential smoothing method	Consumption	International Energy Agency	1993 to 2007	Yearly	Romania	MAPE, MAE, MSE
[67]	Adaptive Residual Compensation	Generation	global energy forecasting competition	2004 to 2014	Hourly	USA	RMSE, R-square, CRPS
[39]	FFNN	Consumption	ASHRAE, library building located in Hangzhou, East China	Sep 1989 to Feb 1990	Hourly	China	MAPE
[40]	FFNN and Bayesian regularization algorithm	Consumption	Building management system	Jul ​2012	15-minute	N/A	MBE, RMSE
[41]	RNN and CNN	Consumption	A City in North China	Feb 2010 to Dec 2012	Hourly	China	MAPE, MAE
[45]	LSTM	Consumption	Individual household electric power consumption	Dec 2006 to Nov 2010	Hourly, and sub-hourly	USA	RMSE
[55]	ARIMA	Generation	Building in Reese Research Center	Nov 2017 to Nov 2018	Monthly	USA	MAPE
[59]	WT, LSTM, SAE	Generation	Energy Information Administration	Jan 1997 to Dec 2022	Monthly	USA	MAE, RMSE, U1, U2
[60]	Ensembled ANN	Generation	Federal Institute of Southern Minas Gerais State	May 2017 to Apr 2019	Weekly	Brazil	MAPE
[61]	ARIMA and ANN	Generation	National Climate Data Center	Jan 2014 to Oct 2019	Daily	Korea	RMSE, MAPE
[3]	LSTM	Generation	Turkish Electricity Transmission Corporation	Jan 2016 to Dec 2019	Daily	Turkey	RMSE, MAE MAPE
***This study***	***LSTM, ARIMA, VAR*** ***LR, Lasso, Ridge, ElasticNet*** ***HuberRegressor, Lars,*** ***LassoLars, PassiveAggressiveRegressor,*** ***RANSACRegressor,*** ***SGDRegressor, and RapidMiner***	***Consumption and Generation***	***RENeW Nexus project***	***Aug 2018 to Apr 2019***	***Hourly***	***Australia***	***RMSE, MAE***

## 3 Time series forecasting models and evaluation metrics

In this section, a discussion is provided on the classical (statistical) and AI-based models which are incorporated in the development of the prediction models in this study. Those models are typically used to forecast the energy consumption and generation of buildings, and implemented through four main stages, namely; data acquisition, data pre-processing, model training, and model testing [68]. Further, certain machine learning tools and software platforms will be presented followed by a discussion on key metrics to evaluate the performance of the time series forecasting model.

### 3.1 Classical time series forecasting models

#### 3.1.1 Univariate and multivariate classical models

Classical time series forecasting models are designed to focus on linear relationships where many of these techniques rely on decomposing time series to three main components; (i) *Trend*: indicates the increase/decrease of data; (ii) *Seasonality*: inferring an iterating pattern in a particular time interval; and (iii) *Noise*: refers to irregular components of data samples [69]. There are various classical time series forecasting models to handle either or both univariate (a single variable) and multivariate (Multiple variables) time series problems.

**Autoregressive Integrated Moving Average (ARIMA):** ARIMA refers to a particular form of regression-based models which presume that time series values are continuous measures [70]. ARIMA is commonly used to be fitted to the time series dataset to gain a better fathom of the dataset and to forecast future points in the examined time series problem. ARIMA models are widely used due to their extensive harness of sophisticated statistics that permit effective determination of the embodied parameters, as well as inclusive valuation of their suitability [71]. ARIMA is used to model stationary and non-stationary time series datasets, and it is mainly composed of two models; Autoregressive Models (AR): uses observations inferred from preceding time steps in the series to feed a regression linear function, thereby forecasting the value at the next time step; Moving Average (MA): uses the past forecasting errors instead in the regression linear function. ARIMA also includes a pre-processing step (Integration (I)) to make the series stationary by conducting a differencing step. Hence, any typical ARIMA model should identify three main parameters; AR(p),MA(q),andI(d), thus the notation of ARIMA can be defined as ${\text{ARIMA}}_{\left(\text{p},\text{d},\text{q}\right)}$. These parameters formulate the forecasting equation of an ARIMA model as follows:

(1) $$x_{t}-\phi_{o}-\phi_{1}x_{t-1}-\phi_{2}x_{t-2}-\cdots -\phi_{p}x_{t-p}=a_{t}+\theta_{1}a_{t-1}+\theta_{2}a_{t-2}+\cdots +\theta_{q}a_{t-q}$$

where $x_{t}$ is the observed output at time t, and $a_{t}$ is the error term at time t.

**Seasonal Autoregressive Integrated Moving-Average (SARIMA):** Seasonal ARIMA or SARIMA is a special form of ARIMA model that is used mainly to model a wide range of seasonal data. This is attained by incorporating an additional seasonal parameter to handle a period of seasonality [72]. SARIMA, therefore, has its own configuration and new different hyperparameters are introduced as follows; (i) P: Seasonal autoregressive order. D: Seasonal difference order; Q: Seasonal moving average order; and m: The number of time steps for a single seasonal period (for example, m = 4 of data is quarterly). The notation of SARIMA can be described as ${\text{SARIMA}}_{\left(\text{p},\text{d},\text{q}\right)}{\left(\text{P},\text{D},\text{Q}\right)}_{\text{m}}$ and can be formulated as:

(2) $$\begin{array}{l} \left(1+\sum_{i=1}^{p}\phi_{i}L^{i}\right)\left(1-\sum_{j=1}^{P}{\text{Φ}}_{j}L^{j\times s}\right){\left(1-L\right)}^{d}{\left(1-L^{s}\right)}^{D}x_{t}\\ \;=\left(1+\sum_{i=1}^{q}\theta_{i}L^{i}\right)\left(1+\sum_{j=1}^{Q}{\text{Θ}}_{j}L^{j\times s}\right)a_{t}\\ y_{t}={\left(1-L\right)}^{d}{\left(1-L^{s}\right)}^{D} \end{array}$$

where $x_{t}$ is the original non-stationary output at time $t,\;y_{y}$ is the stationary output at time t., and $a_{t}$ is the error term at time t.

**Vector Autoregression (VAR):** VAR is used commonly with multiple parallel time series (eg. multivariate time series). It is a generic form of AR for predicting a vector of time series. Hence, it includes one equation for each variable in the model, then VAR generates a forecast for each variable in a repetitive way [72]. VAR model has proven ability to fit several time series problems, this includes analysing a dissimilar number of variables to infer the dynamic relationships among them [73], or investigating whether a certain variable might affect forecasting different variables. In other words, each designated variable is a linear function of previous lags of itself as well as previous lags of other variables. The typical equation for a VAR model can be written as:

(3) $$Y_{t}=\alpha +\beta_{1}Y_{t-1}+\beta_{2}Y_{t-2}+\cdots +\beta_{p}Y_{t-p}+\epsilon_{t}$$

where α is a constant, $\beta 1,\beta_{2}$ to βp are the coefficients of the lags of Y until order p which indicates the number of p-lags of Y used, and $\epsilon_{t}$ indicates the white noise.

#### 3.1.2 Multi-step forecast with linear algorithms

Time series problem is commonly addressed through forecasting one value (observation) in the future (i.e. one-step prediction). However, conducting time series analysis can also include predicting multiple values in the future (i.e., multiple-steps prediction). For example, instead of predicting the temperature of the next day (one-step), forecasting the temperature for the next week (seven-steps) is also valid with multi-step time series. This study will focus on one-step time series forecasting and will leave multi-step forecasting to future work. Python's scikit-learn library^1^ offers a set of generalized linear regression-based models that can be incorporated with one-step and multi-step time series forecasting problems. These include LinearRegression, Lasso, Ridge, HuberRegressor, to name a few.

### 3.2 Deep learning - Long Short-Term Memory (LSTM)

Long Short-Term Memory networks - commonly called "LSTMs" - are a special kind of *Recurrent Neural Network* (RNN), capable of learning long-term dependencies. It was first introduced by Hochreiter & Schmidhuber [74], revised, and circulated by many research fellows at the following work [75, 76, 77]. LSTM layer uses the concept of numerous hidden state types to alter the quantity of information kept across so called *states*. This can be used for instance when working on sequential data (i.e., time-series or text), since the hidden states can store a given amount of information from previous states beyond the just handled one. This means that for example, a connection between the first word of a long text and the last word can be created even though the complete paragraph is quite long. To account for the importance of in-between states, LSTMs use mechanisms to adjust the importance and amount of influence a hidden state has for the current calculation [78].

**Figure 1:**
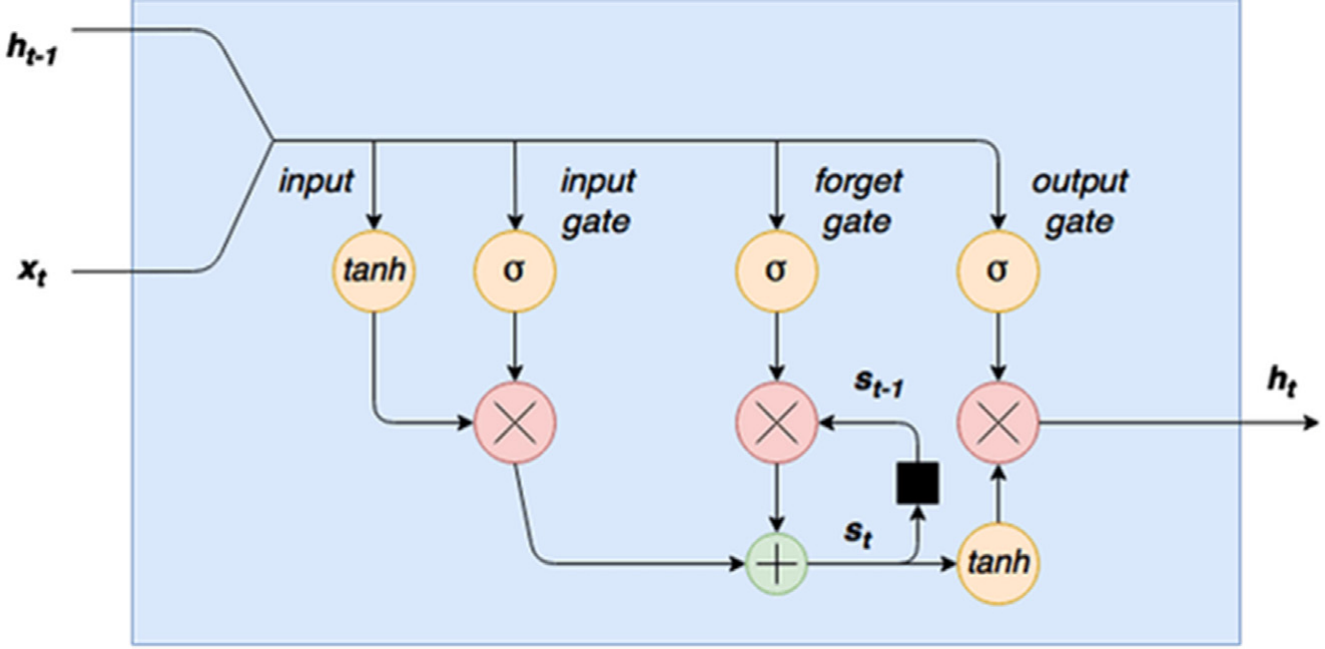
LSTM cell architecture [79].

Figure 1 portrays the inner cell diagram of an LSTM Network. LSTM preserves a hidden vector, h, and a memory vector, m, where the state updates and outputs are controlled at each step, respectively. The computation at each time step is formulated as the following:

(4) $$g^{u}\;=\;\sigma \left(W^{u}h_{t-1}\;+\;I^{u}x_{t}\right)\;$$

(5) $$g^{f}\;=\;\sigma \left(W^{f}h_{t-1}\;+\;I^{f}x_{t}\right)$$

(6) $$g^{o}\;=\;\sigma \left(W^{o}h_{t-1}\;+\;I^{o}x_{t}\right)$$

(7) $$g^{c}\;=\;tanh\left(W^{c}h_{t-1}\;+\;I^{c}x_{t}\right)$$

(8) $$m_{t}\;=g^{f}\;⊙m_{t-1}+\;g^{u}\;⊙g^{c}$$

(9) $$h_{t}\;=tanh\left(g^{o}\;⊙m_{t}\right)$$

Where $g^{u}$ is the activation vector of the *input* gate, $g^{f}$ is the activation vector for the *forget* gate, $g^{o}$ is the activation vector of the *output* gate, and $g^{c}\;$ is the activation vector of the *cell state* gate. $h_{t}$ is the hidden state vector of the LSTM unit, the logistic sigmoid function is indicated by σ, the elementwise multiplication is embodied by ⊙. $W^{u},\;W^{f},\;W^{o},\;W^{c}\;$ represent the recurrent weight matrices. Finally, $I^{u},\;I^{f},I^{o},I^{c}$ notations indicate the projection matrices.

The ongoing incorporation of deep learning approaches is due to their technical capabilities to learn long-term dependencies as well as nonlinear characteristics that are captured in electric data which commonly leads to obtain accurate forecasting results; thus they overshadow other classical or statistical machine learning models [80, 81]. Further, data in an energy forecasting problem might embody a strong periodicity (i.e., oscillates or fluctuates over time) [82]. This can be observed in electricity consumption that commonly peaks at a certain time of the day. This periodicity can be also perceived seasonally (i.e., monthly, yearly, etc.). The literature also verifies the utility of deep learning models in other metrics including evaluation errors, prediction accuracy, and robust generalisation ability [83, 84, 85].

### 3.3 Data science tools

**Figure 2:**
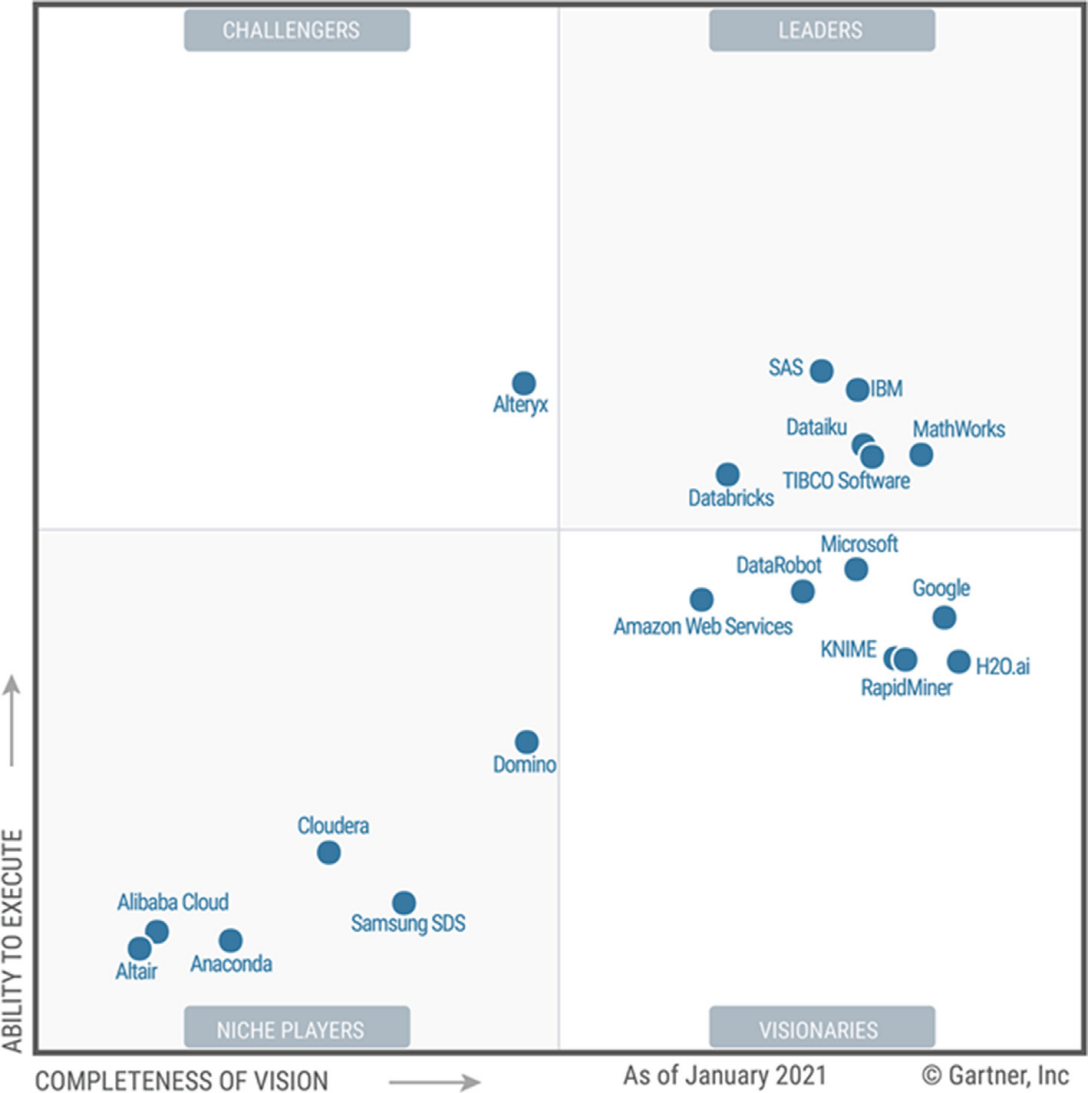
Gartner magic quadrant for data science and machine learning (2021).

With the growing interest in data science and machine learning, the intense rivalry amongst software companies has led those companies to develop cross-platform tools and software systems that are used for conducting machine learning and data mining tasks with minimum efforts. Gartner in their 2021 Magic Quadrant for Data Science and Machine Learning Platforms [86] has placed seventeen distinguished software companies into four different forms of technology providers. As depicted in Figure 2, RapidMiner is amongst four competing companies which are positioned on the *Leader* quadrant for being drivers for transformation.

RapidMiner™ has been incorporated in this study as an alternative rapid approach for conducting time series forecasting. The aim is to examine the results of the conducted experiments using this software platform, thereby providing another benchmark comparison with the obtained results of classical and deep learning-based forecasting techniques.

### 3.4 Metrics for models performance evaluation

This study uses a set of evaluation metrics to measure the performance of the implemented prediction module. The following metrics are used to compare the performance of the model for forecasting energy consumption and generation: (i) Root-Mean-Square Error (RMSE): is used to measure the accuracy of model prediction performance. It is commonly used to calculate standard deviation of the prediction errors (residuals); (ii) Mean Square Error (MSE): provides an indication on how well the regression model is by computing the distance between the data point and the regression line; and (iii) Mean Absolute Error (MAE): it is essentially the mean of the absolute differences between forecasted and actual values.

These metrics can be defined through the following formulas:

(10) $$MSE=\frac{1}{n}\sum_{i=1}^{n}{\left(F_{i}-O_{i}\right)}^{2}$$

(11) $$RMSE=\sqrt{\frac{1}{n}\sum_{i=1}^{n}{\left(F_{i}-O_{i}\right)}^{2}}$$

(12) $$MAE=\frac{1}{n}\sum_{i=1}^{n}\left|F_{i}-O_{i}\right|$$

where $F_{i}$ = forecasted value, $O_{i}$ = the actual value, and n = number of data samples.

## 4 Models development

In this section, we discuss various experiments carried out to develop models for forecasting energy consumption and generation. The experiments that incorporate classical and ML techniques were developed using Python. *Keras*^2^ is also used as an efficient deep learning-based python library. Another set of experiments was also implemented incorporating the RapidMiner™ software platform.

### 4.1 Dataset description and Features Selection

The energy data are collected from August 2018 to Apr 2019 (9 months) at fine time resolution down to 5 s and transmitted through the Internet of Thing network. Amazon Web Services (AWS), a Virtual Private Cloud (VPC), is used to store energy data. The data comprises energy import from the grid, energy export to the grid, energy generation from installed rooftop PV, and energy consumption in households. This paper aims to build forecasting models to predict both energy generation and consumption. The dataset also comprises daily temperature observations collected by the Bureau of Meteorology for Perth, Western Australia^3^.

**Datasets Pre-processing:** a set of data pre-processing and feature engineering steps were followed before conducting experiments: (1) the energy consumption and generation data were aggregated from different sites and transformed from data with 5 s intervals into data with one-hour intervals. The aggregation of all power consumption and generation for all sites was carried out due to the fact that forecasting accuracy commonly declines as the level of aggregation decreases [87]. (2) Energy consumption and generation dataset were integrated with the temperature data obtained from the weather dataset. (3) The null/missing values in the dataset were identified and imputed. (4) A new feature to represent the day of the week is created.

**Figure 3:**
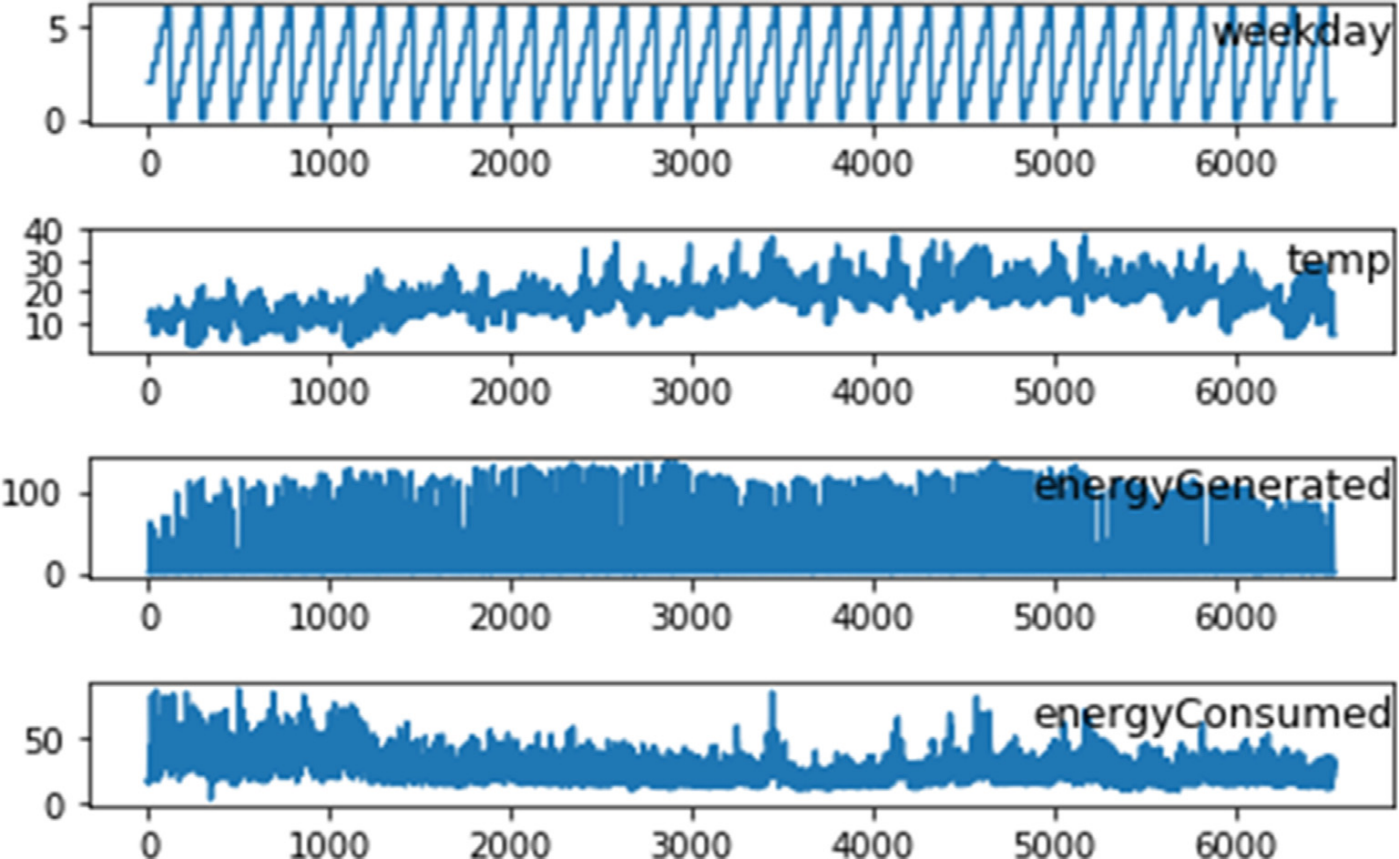
Line plot of energy generation and consumption dataset.

**Features Selection:** The resultant dataset comprises three different variables which are selected due to their direct effect on energy generation and consumption. Those include day of the week (Monday to Sunday), outdoor temperature (⁰C), and the hour of the day (0–23). Table 2 shows some statistics of the processed datasets and Figure 3 illustrates a diagram of three subplots showing temperature data, energy consumption and energy generation during the whole 6120 h period (≈9 months).

**Table 2:** Dataset description.

	Weekday	Temp	Energy Generated	Energy Consumed
mean	3.00	17.46	31.38	29.25
std	2.00	6.35	41.62	12.13
min	0.00	2.80	0.00	3.27
25%	1.00	13.30	0.04	20.21
50%	3.00	17.30	2.55	27.75
75%	5.00	21.20	62.64	35.07
max	6.00	38.00	136.29	88.24

**Figure 4:**
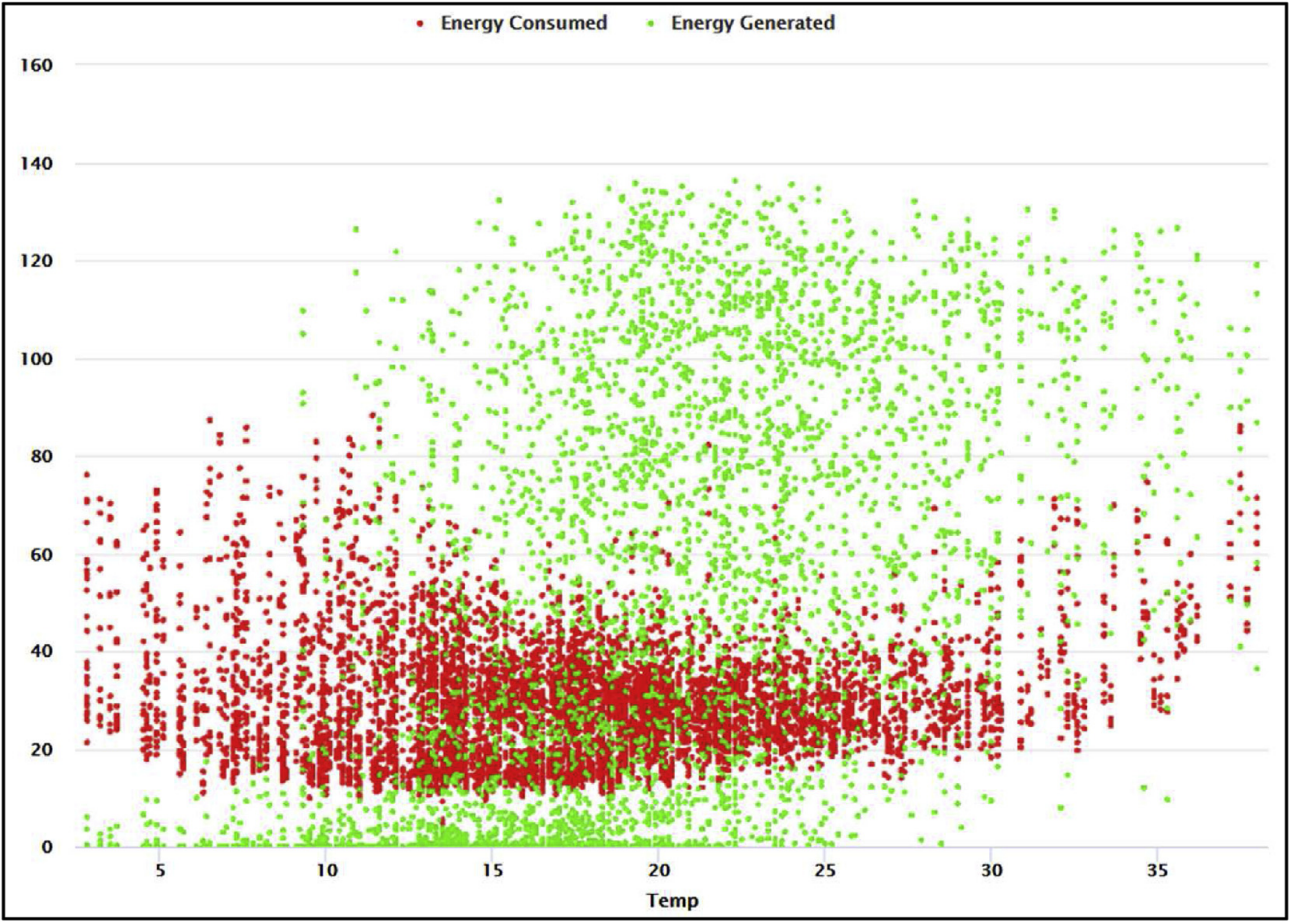
The correlation between temperature and energy consumption and generation.

Figure 4 depicts the correlation between temperature and energy (generated and consumed). It is evident that energy generation and consumption are highly dependent on temperature. People tend to consume much energy on hot days (for cooling) and cold days (for heating). Also, as depicted in Figure 4, there is moderate energy consumption in mild weather conditions. On the other hand, renewable energy production surges as temperature rises, thereby augmenting supplied energy (mix energy) to the smart grid.

**Figure 5:**
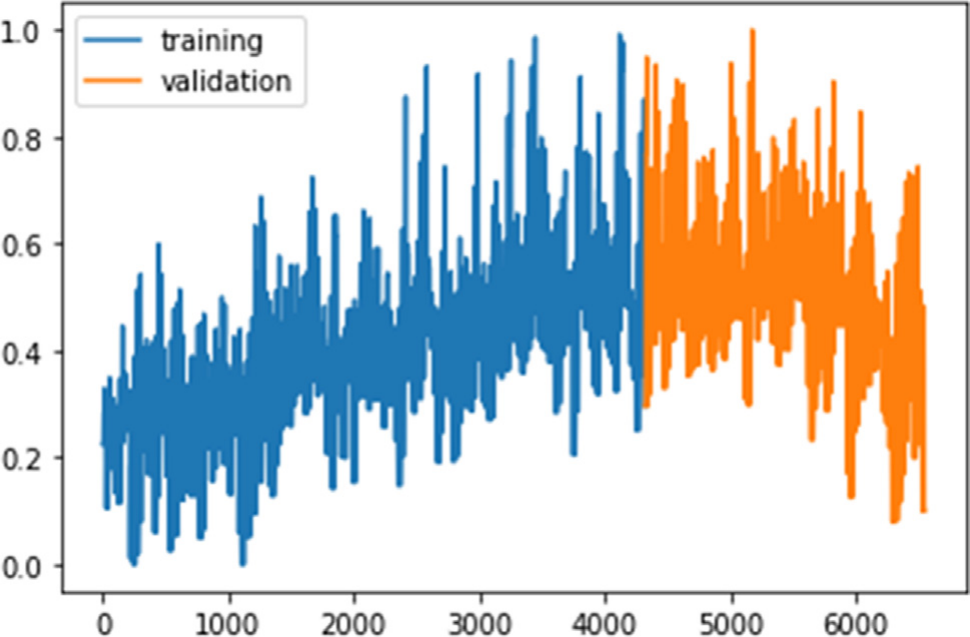
Training and testing datasets.

The dataset from the beginning of Aug 2018 to the end of Feb 2019 was used in the model training process, and the remaining dataset of Mar and Apr 2019 were used to test and evaluate the model performance as depicted in Figure 5.

### 4.2 Multivariate energy consumption and generation forecasting using deep learning

#### 4.2.1 Dataset preparation

**Normalization and reshaping:** incorporating LSTM for time series problem necessitates a need to normalize the dataset and to transform the dataset to a supervised learning problem (i.e., dataset is split into a set of variables/features and an output label). This involves learning from the previous data points in the series to forecast the next value in the future, thereby, the sequence of observations inferred from the series should be transformed as feature variables from which LSTM can learn. Based on suggestions provided by [88], we frame our time series problem as forecasting the energy consumption/generation at the current time (t) given the temperature, week of the day, and energy consumption/generation at the former time step.

**Figure 6:**
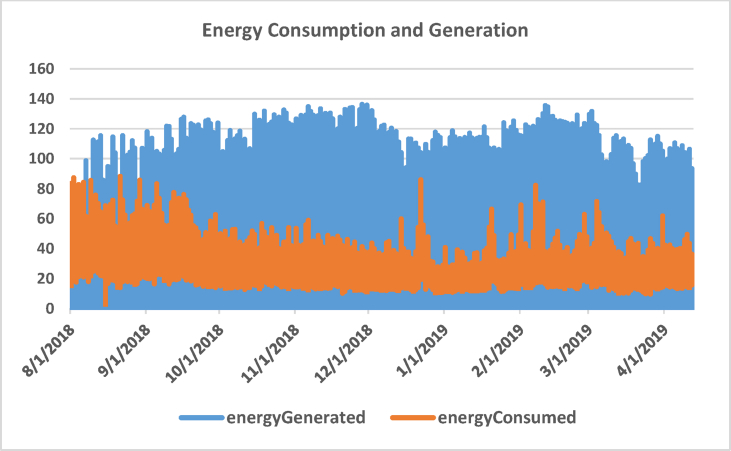
Energy consumption and generation over time.

**Data Stationary:** the next step is to ensure that the dataset is stationary which facilitates model development. This can be obtained by ensuring that there is no regular increase or decrease in the data over time (i.e., data is time-independent). Figure 6 portrays the relationship between both energy consumption and generation over time. It is evident that both energy generation and consumption do not depict any explicit trend over time, this data is stationary.

**Time Series Scaling:** LSTM, like any other neural network, requires the dataset to be scaled to fit its activation function. The default activation function of LSTM is tanh function, its output values are in the range of [−1,1] which is a favorable range for time series data. Hence, scaling was applied to the dataset, and min/max coefficients were calculated on both the training and testing dataset.

#### 4.2.2 LSTM network model design

**Grid Search Hyperparameters Settings:** constructing a neural network is not a conventional task; the selection amongst dissimilar parameters for LSTM setting consumes time and effort, but it is crucial to attain high-quality prediction model. This is because the forecasting performance of a developed model might vary based on dissimilar hyperparameter settings provided, this is due to the stochastic nature of LSTM algorithm where different batch sizes for example lead LSTM to learn differently each time it maps inputs with outputs. Therefore, hyperparameter optimization technique such as *grid search* was incorporated in this study to examine various LSTM structures of dissimilar hyperparameters settings. In particular, grid search comprises building a distinct LSTM for a combination of different parameters to tuning a neural network, thereby finding the optimal settings for building a competent forecasting model. To obtain this, we build a dictionary depicted in Table 3 containing a list of selected parameters with the incorporated selected settings available in the Keras library.

**Table 3:** LSTM's hyperparameters and their settings.

Hyperparameter	Examined Settings
Batch Sizes	2,3
Number of Neurons	2,3
No of Epochs	1000,1500,2000
Optimization algorithms	SGD, RMSprop, Adagrad, Adadelta, Adam, Adamax, Nadam
Activation Functions	tanh, softmax, elu, selu, softplus, softsign, relu, sigmoid,
Losses	mse, mae, mape, logcosh
Dropout Rate	0.0, 0.1, 0.2, 0.3, 0.4, 0.5, 0.6, 0.7, 0.8, 0.9

### 4.3 Models development using RapidMiner™

**Figure 7:**
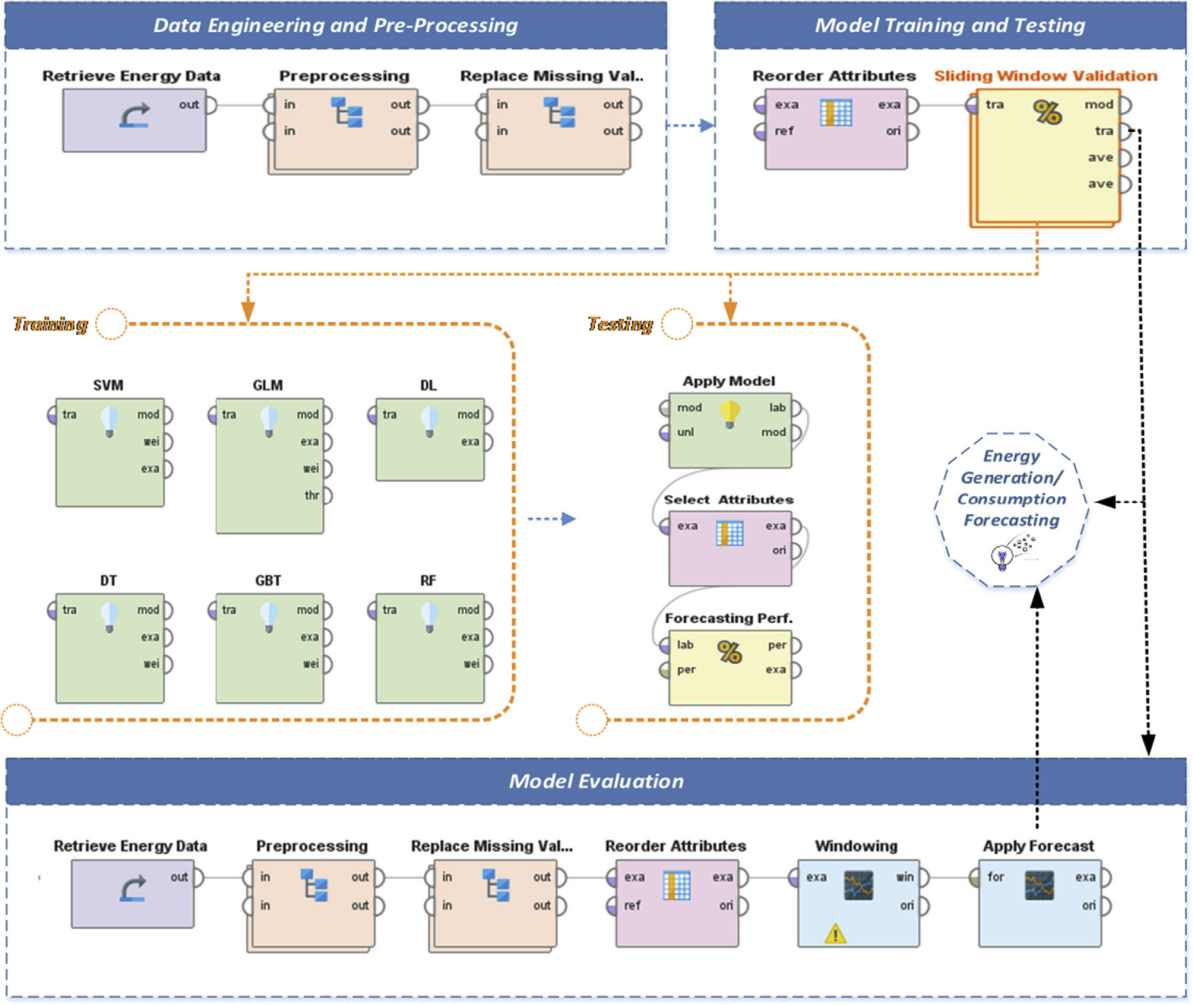
Model process development using RapidMiner.

RapidMiner™ was used to forecast both energy consumption and generation using diverse embodied machine learning-based statistical techniques, namely; Generalised Linear Model (GLM), Deep Learning (DL), Random Forest Tree (RFT), Gradient Boosted Tree (GBT), Decision Tree (DT), and Support Vector Machine (SVM). Figure 7 depicts the model development process incorporating RapidMiner studio.

As depicted in Figure 7, the developed framework using RapidMiner™ comprises the following main stages with their embodied operators:

(i) **Data Engineering and Pre-processing:** in this phase, the dataset of energy consumption and generation passed through the set of data acquisition and pre-processing operators where the dataset is initially loaded with its metadata to the RapidMiner Object. Dataset then is regularised, unified, and mapped. In particular, pre-processing operator alters the type of the numerical attributes to real (float) data type. It also maps all values of these attributes to real values. Dataset Engineering in RapidMiner also includes replacing the missing value with the minimum, maximum or average value of the designated attribute - average value is chosen in our case. Finally, the dataset was split into three different subsets (40% Training, 20% Testing, and 20% Validation)
(ii) **Model Training and Testing:** after the data engineering and pre-processing phase, the dataset passed through the “Reordering” operator that allows reordering the regular features of the dataset. RapidMiner allows to customize the reordering operator where ordering can be done alphabetically (including Regular Expressions) or with a reference – the default alphabetical ordering was used. Then, the dataset passed through the “Sliding Window Validation” operator. This operator comprises a set of sub-operators encapsulating the sliding windows of both training and test datasets and also is responsible to measure the performance of a prediction operator. The average of the performance measurements is computed after the window is moved over the dataset. Further, as depicted in Figure 7, the Sliding Window Validation operator embodies the set of all Machine Learning modules incorporated for training in this experiment alongside delivering a list of performance values based on the performance settings indicated for each Machine Learning technique.
(iii) **Model Evaluation:** by completing the previous phase, the final performance of the various machine learning algorithm is tested through several experiments to infer the optimal hyperparameter settings for each module. In Model Evaluation an assessment was carried out to see how well the model is able to generalize. This is through using data samples that were not used to build the model, the aim is to provide an unbiased evaluation of final model effectiveness [89], by means of evaluating the final hypothesis by an independent evaluation [90].

As mentioned previously, the aim of this study is to provide an efficient forecasting platform to predict both renewable energy generation and energy consumption in households. The next section presents several experiments conducted to find the optimal model for energy generation and consumption.

## 5 Experimental results and discussion

### 5.1 Forecasting results based on LSTM model

#### 5.1.1 Baseline model of performance

**Figure 8:**
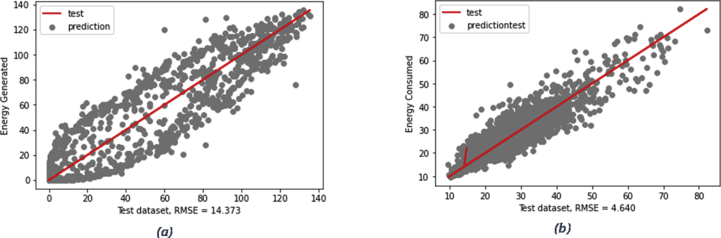
Persistence baseline with the resultant RMSE on energy generation (a) and energy consumption (b).

Building a baseline model for a time series problem (or generally any machine learning problem) is crucial as it establishes a benchmark comparison with the developed model. One of the most frequently used baseline models for time series problems is the *persistence algorithm* which essentially forecasts the value of the next time step (t+1) using the inferred value from the previous time step (t-1). Evaluating this baseline model was carried out using the *rolling forecast* or *walk-forward* validation approach as an alternative approach to cross-validation. Cross-validation could not be used in a time series problem as it neglects the fact that temporal factor links all observations of the time series dataset, thus samples are time-dependent. This differs from a typical machine learning problem where samples are independent with commonly no explicit relationships. Figure 8 illustrates the actual and prediction values from both energy consumption and generation tests datasets, also RMSEs values for both baseline models are indicated which will be used in the experiments discussed next section.

#### 5.1.2 Experimental results on energy generation and consumption

The experiments were initiated by developing a model to forecast energy generation and consumption. Grid search optimization technique was used, thereby various LSTM network designs were constructed and tested to infer the optimal structure with the best hyperparameter settings. Table 4 presents the optimal settings for each task obtained by conducting a grid search for the LSTMs’ hyperparameters. Table 4 also illustrates the values of key metrics inferred from the conducted designated experiment for both tasks (i.e., Energy Generation and Energy Consumption).

**Table 4:** Optimal hyperparameter settings and evaluation metric of energy generation and consumption forecasting using LSTM model.

Task	Hyperparameters							Evaluation Metric	
	No. epochs	No. neurons	Batch size	optimizer	Dropout rate	activation	loss	RMSE	MAE
Energy Generation	2000	3	2	Adam	0.2	tanh	mae	0.5654	0.329
Energy Consumption	2000	3	3	SGD	0.2	relu	mae	0.3273	0.2410

**Figure 9:**
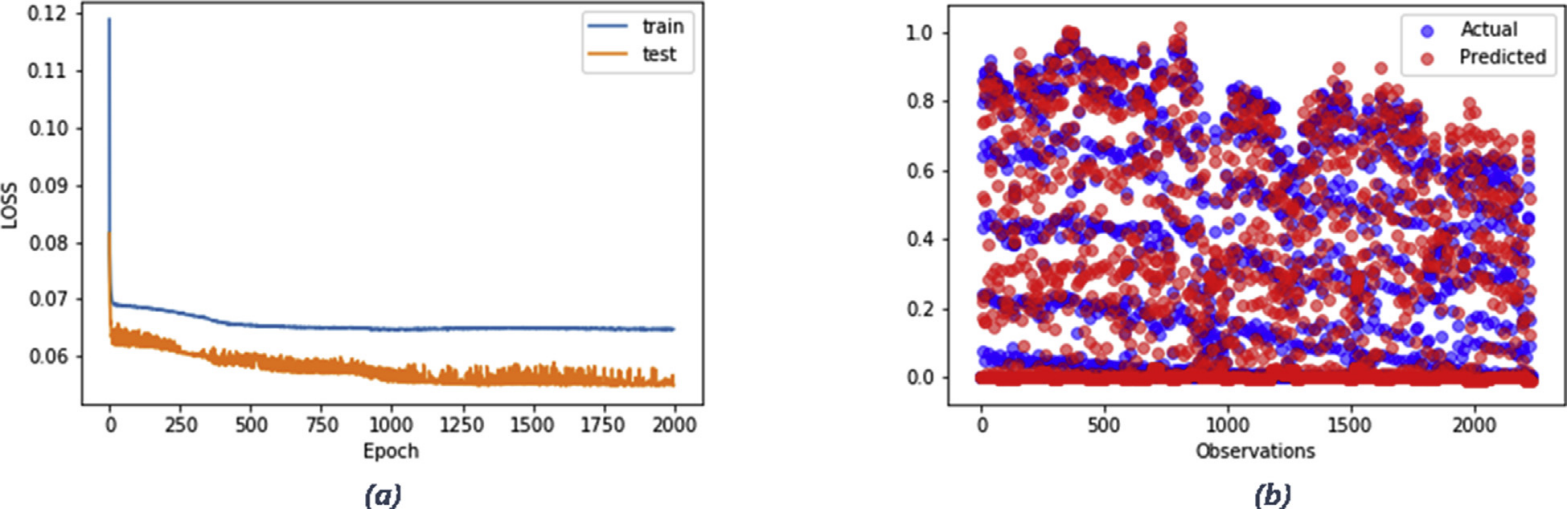
Experiment result of energy generation forecasting using LSTM model: a) a plot of train and test loss, b) a plot of actual vs prediction values.

**Figure 10:**
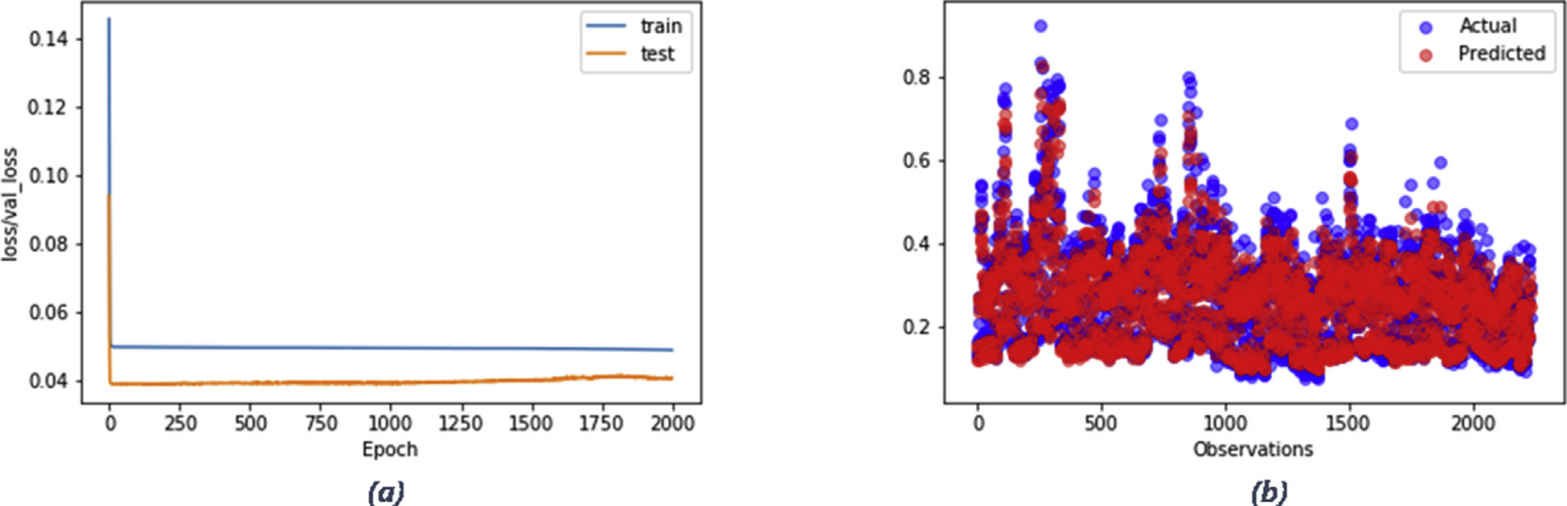
Experiment result of energy consumption forecasting using LSTM model: a) a plot of train and test loss, b) plot of actual vs prediction values.

As depicted in Table 4, the developed model has proven the ability to outperform the baseline model as the resultant RMSE is lower than the inferred value from the persistence baseline model for both tasks. Figure 9 and Figure 10 portray the reported loss and validation loss per epoch obtained from training the LSTM models (experiments of the lowest RMSEs) designed for energy generation and consumption forecasting respectively. The loss/validation_loss values were computed on training and validation datasets (summary of the errors inferred by each example in training and validation sets at successive epochs) and the plots show that both models designed for the two tasks are skill on these two datasets.

#### 5.1.3 A comparison with classical time series forecasting algorithms

Another thread of experiments was implemented to compare the deep learning LSTM technique with several classical and linear algorithms. In fact, those models had been implemented initially to scrutiny their performance before conducting any further experiments. This is due to the claim that the classical time series-based approach might outweigh other sophisticated statistical deep learning approaches [91]. However, as depicted in Table 5, the experiment on more than ten classical and linear-based algorithms to forecast both energy generation and consumption did not report high performance in terms of RMSE metric and did not outweigh the LSTM deep learning model for both tasks.

**Table 5:** RMSEs on using classical and statistical time series algorithms for Energy Consumption (EC) and Energy Generation (EG).

Linear/Classical Algorithm	RMSE		MAE	
	EG	EC	EG	EC
ARIMA	4.256	4.539	3.325	3.482
VAR	6.452	5.983	4.254	4.198
LinearRegression	2.110	2.082	1.568	1.568
Lasso	2.045	2.023	1.770	1.770
Ridge	2.109	2.082	1.569	1.568
ElasticNet	2.042	2.019	1.765	1.765
HuberRegressor	2.500	2.455	1.743	1.742
Lars	2.110	2.082	1.568	1.568
LassoLars	2.045	2.023	1.771	1.770
PassiveAggressiveRegressor	6.133	6.088	2.41	2.409
RANSACRegressor	2.464	2.421	1.391	1.389
SGDRegressor	2.103	2.137	1.612	1.592

### 5.2 Forecasting results using RapidMiner™

The experiment on RapidMiner™ was carried out based on the methodology discussed in section 4.3 and the approach provided. Table 6 presents a summary of the key parameters and their settings for the experiment conducted using RapidMiner platform. These settings comprise values inferred by RapidMiner using their auto model to fit the designated regression problem.

**Table 6:** Selected parameters settings.

Parameter	Description	Value
**Windowing**		
*Window size*	number of values per window	20
*Step size*	Size between the first values of two successive windows	1
*Horizon size*	The number of values taken as the horizon (i.e. time points).	24

**Machine Learning Models**		
**Gineralised Linear Model (GLM)**		
*Solver*	For optimisation	IRLSM
*maximum number of threads*	Controls parallelism level of building model	1
*number of lambdas*	Controls the amount of applied regularization	30

**Artificial Neoral Network (ANN)**		
*Activation function*	Function used by neurons in the hidden layers	Rectifier
*No. of hidden layer*	Number of hidden layers in the model	2
*No. of neurons per layer*	Size of each hidden layer	50
*Epochs*	Iteration times over dataset	10
*L1*	Regularization (absolute value of the weights)	1.0E-5
*L2*	Regularization (sum of the squared weights)	0.0
*Loss function*	loss (error) function	Auto

**Random Forest Tree (RFT)**		
*No. Trees*	Number of random generated trees	20
*Criterion*	On which attribute will be split	least_square
*Max_depth*	Depth of the tree	7

**Gradient Boosted Tree (GBT)**		
*No. Trees*	Number of generated trees	150

Decision Tree (DT)		
*Criterion*	On which attribute will be split	least_square
*Max_depth*	Depth of the tree	15

**Support Victor Machine (SVM)**		
*Kernel Type*	Kernel Function used in the model	Radial
*Kernel gamma*	SVM kernel parameter gamma	1.0000000000000007
*kernel cache*	size of the cache for kernel evaluation (MB)	200
*C*	SVM complexity constant	1000

RapidMiner computes the performance based on 20% of the unseen dataset. This dataset is embedded for a multi-hold-out-set validation where average performance is computed for seven dissimilar subsets. Table 7 shows the performance metrics of six different machine learning algorithms. As depicted in the table, Gradient Boosted Tree obtains the best performance in both evaluation metrics amongst all techniques to forecast both power consumption and power generation. This is commonly due to the strong scalability and regularization quality of the GLM amongst other techniques [92, 93].

**Table 7:** Performance metrics for Energy Consumption (EC) and Energy Generation (EG).

	GLM		ANN		RF		GBT		DT		SVM	
	EG	EC	EG	EC	EG	EC	EG	EC	EG	EC	EG	EC
RMSE	10.253	9.76	12.742	7.353	10.25	8.254	**8.275**	**4.342**	12.291	7.976	14.383	6.164
MAE	8.796	7.211	8.687	5.331	8.69	6.011	**4.214**	**2.918**	5.824	5.544	8.298	4.258

**Figure 11:**
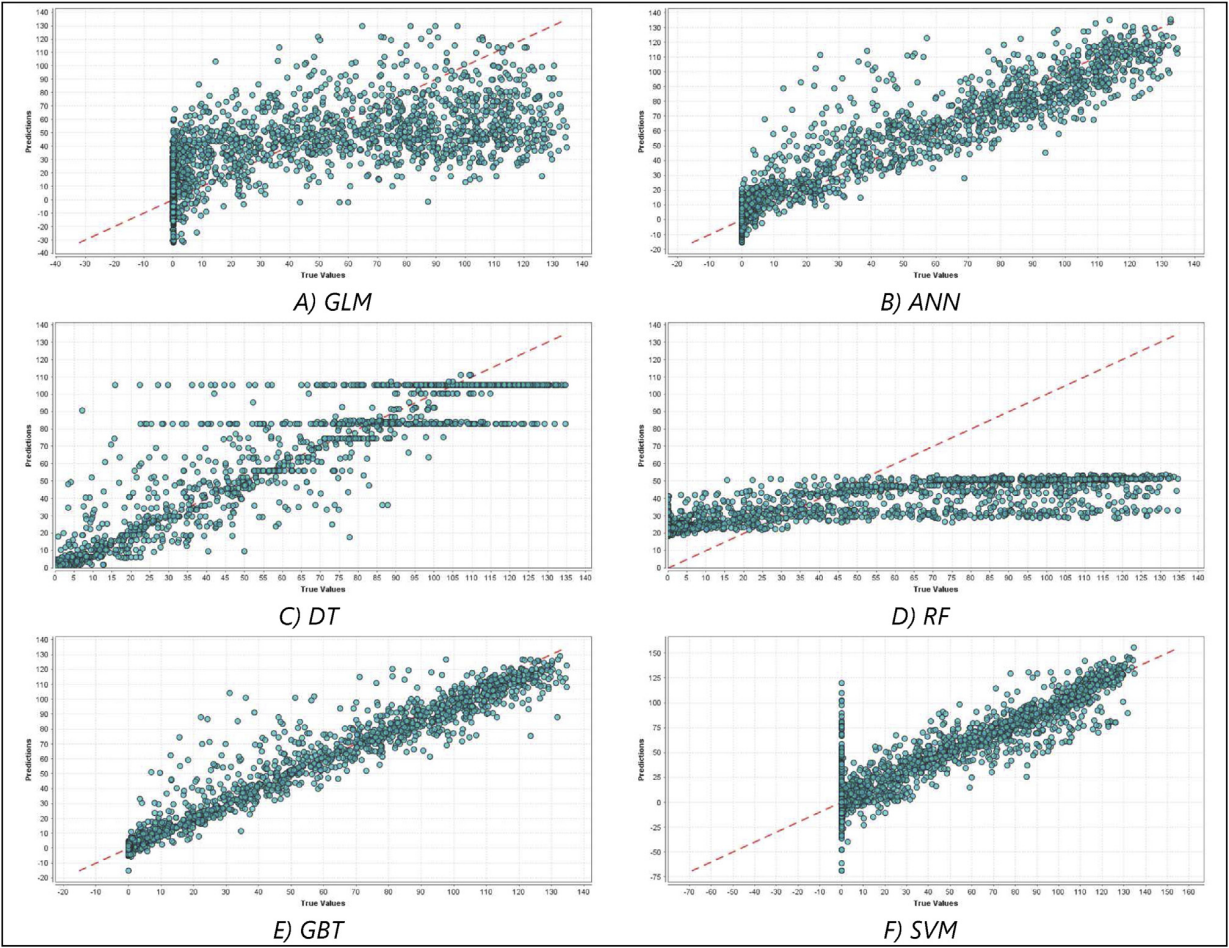
Energy generation prediction charts (the predictions vs. the actual values) of different regression models tested on RapidMiner. A) Prediction chart for GLM Model, B) Prediction chart for ANN model, C) Prediction chart for DT model, D) Prediction chart for RF model, E) Prediction chart for GBT model, and F) Prediction chart for SVM model.

**Figure 12:**
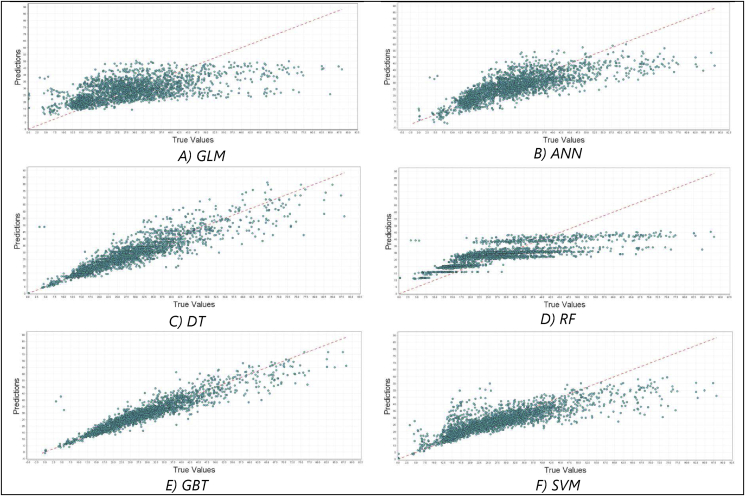
Energy consumption prediction charts (the predictions vs. the actual values) of different regression models tested on RapidMiner. A) Prediction chart for GLM Model, B) Prediction chart for ANN model, C) Prediction chart for DT model, D) Prediction chart for RF model, E) Prediction chart for GBT model, and F) Prediction chart for SVM model.

Figure 11 and Figure 12 portray the predictions plots of the six different regression algorithms tested using RapidMiner on energy generation and energy consumption respectively. These charts show the predictions vs. the actual values for the validation samples. It is evident that Random Forest Tree performance was poor on both tasks, this is due to the fact that RF commonly forecasts an average of unseen training samples since it is not able to extrapolate the normal increasing/decreasing trend in the dataset, thereby does not scale well for time series data.

### 5.3 Aggregated experimental results

**Figure 13:**
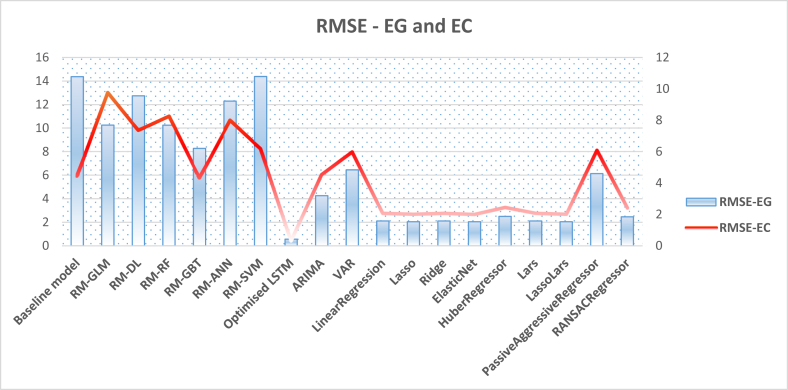
Aggregated RMSE values obtained by all models in forecasting both energy generation and consumption.

**Figure 14:**
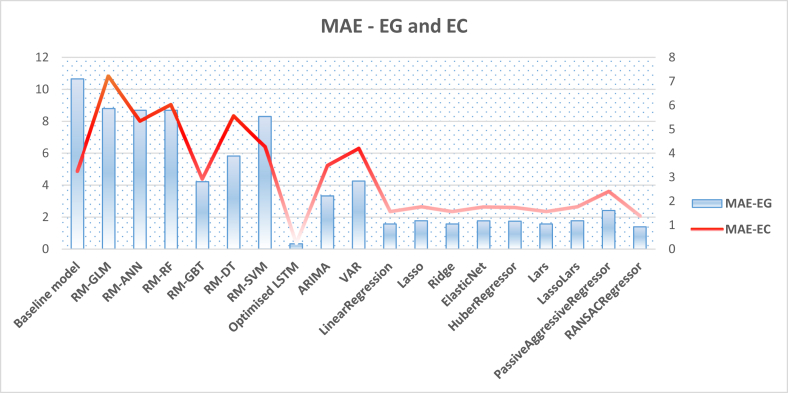
Aggregated MAE values obtained by all models in forecasting both energy generation and consumption.

Previous sections present and discuss the experimental results that are implemented using the baseline model, optimised LSTM, classical models, and RapidMiner embedded machine learning models. To obtain a holistic view, we aggregate the resultant values of the evaluation metrics obtained by all models in two figures, thereby providing better insights. Figure 13 demonstrates the aggregated RMSE values obtained by all models in forecasting both energy consumption and generation experiments, while Figure 14 shows the MAE values obtained in the same designated experiments. These figures verify again the superiority of the optimised LSTM and its utility to provide energy consumption and generation predictions with minimum errors. The next section furnishes a further discussion on these empirical results and cast more light on the significance of the study.

## 6 Discussion, limitations, and future work

In the energy sector, embracing distributed renewable resources is recognised as a potential solution to the climate change problem [94]. Therefore, the energy sector is presently shifting promptly to accommodate sustainable energy transitions driven by forces of technological innovation and advances of digitalisation platforms. Opportunities for distributed energy markets are tremendous and being trialled around the world including pilot projects are being trialled across the world to explore the potential of P2P energy trading, including the RENeW Nexus project in the city of Fremantle, Western Australia [5]. However, to increase operation efficiency (including energy consumption and generation scheduling) as well as make the most efficient energy trading between prosumers and consumers it is imperative to build intelligent systems that are able to estimate energy usage and production in such a decentralised environment. This study attempts to address this issue by developing a correlated array of various classical and advanced AI-based techniques to forecast both energy generation and consumption. The empirical results have concluded with important outcomes that can be summarised as follows.

The first observation is the superiority of the optimised LSTM model in the designated experiments. The abstract structure of the LSTM model enables capturing strong periodicity due to its capacity to maintain the temporal correlation utilising the memory block that is embedded in the recurrent layer [95]. Hence, contextual information represented by the temporal or spatial dependency of data can be attained and well-presented [96]. It is also important to highlight the significance of the hyperparameter optimization process that has been carried out on LSTM to infer the best set of settings so as to fit well the incorporated dataset. The second observation is the poor performance of the autoML tool, namely RapidMiner, and its embedded ML on the designated tasks in comparison to the optimised LSTM as well as the classical models. Figure 11 and Figure 12 provide an evident viewpoint on this inadequacy of RapidMiner's built-in settings to tackle the forecasting problem. Also, this study points to the importance to carry out data preprocessing and preparation to enhance data quality prior to data analytics. This is through data normalisation, reshaping, and scaling as well as ensuring stationarity of data.

This paper attempts to provide an efficient forecasting platform to predict both renewable energy generation and energy consumption in households. However, this study exhibits certain limitations that we hope to address in the future. For example, despite the applicability of LSTM to tackle the designated problem due to its capacity to handle sequence pattern information, LSTM utilises only the attributes given in the training dataset with inadequacy to capture patterns that appear in local and global trends of the time series. Extracting patterns of the data can be attained by implementing a Convolutional Neural Network (CNN) model [81]. Therefore, a promising avenue for future research is to develop a hybrid model incorporating both LSTM and CNN that can enhance forecasting accuracy. Also, bagging and other ensemble machine learning strategies will be investigated. In the same context, other advanced hyperparameter and feature selection strategies including bayesian optimization, random optimization, and evolutionary optimization will be examined. This study utilises one-time step as a forecasting approach. Strategies for multi-step time series forecasting will be scrutinized in future research using large-scale datasets to provide further flexibility, thus forecasting multiple time steps instead of one single time step. Finally, more features/attributes will be incorporated, thereby providing a comprehensive multivariate forecasting approach, for example, by examining behavioral characteristics of households’ residents in terms of their electricity consumption and generation.

## 7 Conclusion

This study presents several conducted experiments to forecast hourly-based energy consumption and generation. Real-time data were collected from smart meters that were installed in residential premises and used to evaluate the efficacy and effectiveness of various well-known classical (statistical) and AI-based models that are used to predict energy consumption and production. This paper furnishes the following contributions (i) A two-fold analytical model to forecast energy consumption and generation in P2P settings is designed and implemented; (ii) an auto-ML model and various classical and advanced AI-based techniques are incorporated, and their utility is evaluated and compared comprehensively; and (iii) to the best of our knowledge, this is the first paper that furnishes a forecasting model for both energy consumption and generation based on real data captured from a P2P grid system in the state of Western Australia.

## Declarations

### Author contribution statement

Bilal Abu-Salih: Conceived and designed the experiments; Performed the experiments; Analyzed and interpreted the data; Contributed reagents, materials, analysis tools or data; Wrote the paper.

Pornpit Wongthongtham: Analyzed and interpreted the data; Contributed reagents, materials, analysis tools or data; Wrote the paper.

Greg Morrison and Kevin Coutinho: Contributed reagents, materials, analysis tools or data; Wrote the paper.

Manaf Al-Okaily and Ammar Huneiti: Analyzed and interpreted the data; Wrote the paper.

### Funding statement

This research did not receive any specific grant from funding agencies in the public, commercial, or not-for-profit sectors.

### Data availability statement

Data will be made available on request.

### Declaration of interests statement

The authors declare no conflict of interest.

### Additional information

No additional information is available for this paper.

## Footnotes

1 https://scikit-learn.org/stable/modules/linear_model.html.

2 https://keras.io/.

3 http://www.bom.gov.au/climate/data/.
